# Cytotoxic efficacy of a novel dinuclear platinum(II) complex used with anti-MUC1 in human breast cancer cells

**DOI:** 10.1007/s11010-014-2018-2

**Published:** 2014-03-18

**Authors:** Agnieszka Gornowicz, Zbigniew Kałuża, Anna Bielawska, Halina Gabryel-Porowska, Robert Czarnomysy, Krzysztof Bielawski

**Affiliations:** 1 Department of Biotechnology, Medical University of Bialystok, Kilińskiego 1, 15-222 Białystok, Poland; 2 Institute of Organic Chemistry, Polish Academy of Sciences, Kasprzaka 44/52, 01-224 Warsaw, Poland; 3 Department of Medical Chemistry, Medical University of Bialystok, Kilińskiego 1, 15-222 Białystok, Poland; 4 Department of Synthesis and Technology of Drugs, Medical University of Bialystok, Kilińskiego 1, 15-222 Białystok, Poland

**Keywords:** Monoclonal antibody anti-MUC1, Cisplatin, Dinuclear platinum(II) complexes, Combined therapy, Breast cancer

## Abstract

Mucin 1 (MUC1) is overexpressed in various cancer cells especially in breast cancer cells. There are known research works on the use of anti-MUC1 antibody with docetaxel in ovarian cancer, but there are no data about combined therapy platinum compounds with anti-MUC1 in breast cancer. The aim of the study was to evaluate the antiproliferative properties of a new dinuclear platinum(II) complex (Pt12) used with anti-MUC1 in human breast cancer cells. The dinuclear platinum(II) complex (Pt12) has been synthesized, and its cytotoxicity with anti-MUC1 has been tested in both MCF-7 and MDA-MB-231 breast cancer cells. In this study, the effects of Pt12 with anti-MUC1 on collagen and DNA biosynthesis in human breast cancer cells were compared to those evoked by cisplatin and cisplatin with anti-MUC1. The mechanism of action of Pt12 with anti-MUC1 was studied employing flow cytometry assessment of annexin V binding assay. It was found that Pt12 with anti-MUC1 was more active inhibitor of DNA and collagen synthesis as well more cytotoxic agent than Pt12 alone and cisplatin with anti-MUC1. Cytotoxicity of Pt12 with anti-MUC1 against breast cancer cells is due to apoptotic cell death as well as necrotic cell death. These results indicate that the use of Pt12 with anti-MUC1 may constitute a novel strategy in the chemotherapy of breast cancer tumors.

## Introduction

Mucin 1 (MUC1) is a type I transmembrane glycoprotein that is expressed on apical membranes in a variety of normal tissues, but aberrantly is overexpressed in various cancer cells especially in breast cancer cells [1, 2]. In addition, MUC1 expression has also been demonstrated in hematological malignancies, such as multiple myeloma, Ki-1 positive B cell, and T-cell limphomas [3]. MUC1 is translated as a single polypeptide that undergoes autocleavage into N-terminal (MUC1-N) and C-terminal (MUC1-C) subunits. MUC1-N contains the highly glycosylated tandem repeats whereas MUC1-C extracellular domain interacts as a ligand with galectin-3 and forms complexes with EGF receptor (EGFR) [4]. Particularly in breast cancer, the MUC1–galectin-3 interaction might have functional roles in transformation and metastasis. Galectin-3-mediated MUC1-C–EGFR binding might elicit EGFR-mediated downstream signaling pathways that are involved in tumorigenesis and cancer cell growth [5]. Furthermore, an interaction between MUC1 on the surface of cancer cells and serum-localized galectin-3 promotes strong adhesion of tumor cells to the endothelial surface, thus promoting cancer cell metastasis [6]. In vitro studies demonstrated that the expression of MUC1 is involved in the invasion and resistance to genotoxic anticancer reagents suggesting that it is closely associated with poor prognosis of patients with breast cancer [7]. Investigators reveal that MUC1 has immense potential as diagnostic or prognostic marker and as therapeutic targets in breast cancer [8]. Monoclonal anti-MUC1 antibody is mouse IgG1 isotype derived from the GP1.4 hybridoma, reacts with the repetitive protein epitope and recognizes highly glycosylated episialin (MUC1) in tumors of epithelial cell origin and large cell anaplastic lymphoma.

Polynuclear platinum complexes constitute a novel class of prospective anticancer agents that have shown some peculiar activities as compared with mononuclear platinum compounds [9–12]. The adducts formed by multi-nuclear platinum complexes are vastly different from the adducts formed by cisplatin [13]. It has been suggested that the distortions induced by these complexes are only weakly recognized by DNA repair proteins. Structurally novel platinum complexes that bind to DNA differently than cisplatin may have distinct cytotoxicity and side effect profiles [10, 12].

**Fig. 1:**
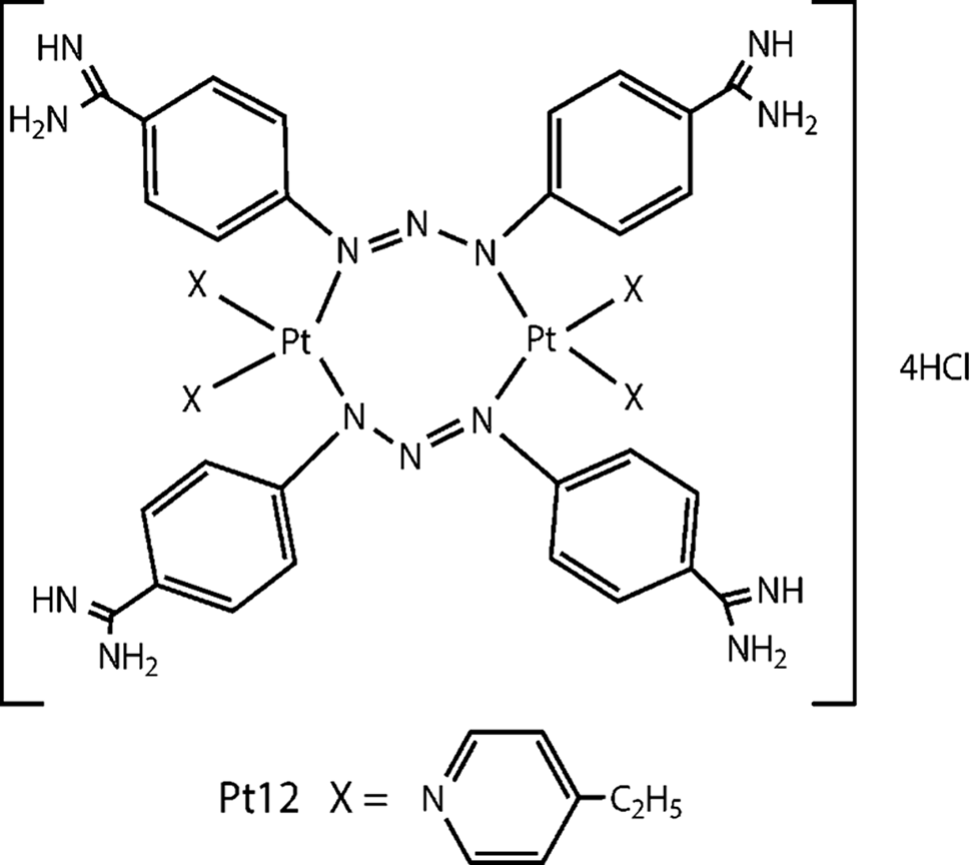
Structure of compound Pt12

The aim of the study was to evaluate the antiproliferative properties of a new dinuclear platinum(II) complex Pt12 (Fig. 1) used together with anti-MUC1 monoclonal antibody in human breast cancer cells and human fibroblasts. We also investigated the proapoptotic properties Pt12 with anti-MUC1 using flow cytometry assessment of annexin V binding, analysis of mitochondrial membrane potential, and defragmentation of DNA by TUNEL assay. The cellular response of human breast cancer cells to Pt12 with anti-MUC1 has been studied using cisplatin as a reference.

## Materials

Dimethylformamide, K2PtCl4, KI, acetone, 4-ethylpyridine, diethyl ether, methanol, ethidium bromide, cisplatin, 3-(4,5-dimethylthiazol-2-yl)-2,5-diphenyltetrazolium bromide (MTT), and monoclonal antibody anti-MUC1 GP1.4 were purchased from Sigma Chemical Co. (USA). Stock cultures of human MCF-7 and MDA-MB-231 breast cancer cells were purchased from the American Type Culture Collection (USA). Dulbecco minimal essential medium (DMEM) and fetal bovine serum (FBS) used in a cell culture were products of Gibco (USA). Glutamine, penicillin, and streptomycin were obtained from Quality Biologicals Inc. (USA). [^3^H]thymidine (6.7 Ci mmol^−1^) was purchased from NEN (USA), and Scintillation Coctail “Ultima Gold XR” from Packard (USA). Sodium dodecylsulfate was received from Bio-Rad Laboratories (USA). FITC Annexin V Apoptosis Detection Kit II and JC-1 MitoScreen Kit were products of BD Pharmigen.

## Physical measurements

The structure of a synthesized compound was confirmed by ^1^H NMR and ^13^C NMR spectra recorded on Brucker AC 200F (Germany) apparatus (^1^H—200 MHz and ^13^C—50 MHz) in deuterated dimethyl sulfoxide (*d*_6_-DMSO). Chemical shifts are expressed in *δ* value (ppm). Multiplicity of resonance peaks are indicated as singlet (s), doublet (d), triplet (t), quartet (q), and multiplet (m). Infrared spectra were recorded on Perkin Elmer Spectrum 100 FT-IR spectrometer (USA) as KBr pellets (4,000–450 cm^−1^). Melting points were determined on Büchi 535 (GER) melting-point apparatus and were uncorrected. Elemental analysis of C, H, and N was performed on a Perkin Elmer 240 analyser (USA) and satisfactory results within ±0.4 % of calculated values were obtained.

## Chemical synthesis of [Pt_2_(4-ethylpyridine)_4_(berenil)_2_]·4HCl·2H_2_O (Pt12)

K2PtCl4 (0.72 mmol) was dissolved in 40 mL of deionized water. KI (7.2 mmol) was added to it, and the reaction mixture was stirred for 30 min. Then, 4-ethylpyridine (1.44 mmol) was added dropwise to the reaction mixture while stirring, to obtain a precipitate, *cis*-[Pt(4-ethylpyridine)_2_I_2_]. The stirring was continued for 60 min, and the precipitate was then collected by filtration. This compound was filtered, then was washed with 30 mL deionized water and 5 mL acetone and dried in vacuum. *Cis*-[Pt(4-ethylpyridine)_2_I_2_] (1.22 mmol) was suspended in 5 mL of an aqueous solution of silver nitrate (AgNO_3_) (2.44 mmol). The reaction mixture was stirred for 24 h at room temperature in the dark. The AgI precipitate was filtered off. Berenil (1.22 mmol) and a solution of 10 % NaCl (5 mL) were added to the filtrate and stirring until a precipitate of [Pt_2_(4-ethylpyridine)4(berenil)2]·4HCl·2H2O formed. Afterward, the product was filtered off and washed with a small amount of diluted HCl, deionized water, methanol, acetone, and ethyl ether and dried under vacuum. Yield: 64 %; yellow powder; mp 208–212 °C; ^1^H NMR (DMSO-*d*_6_) *δ* (ppm): 9.35 (br s, 4H, amidine), 9.00 (br s, 4H, amidine), 8.55 (d, *J* = 7.5 Hz, 8H, Ar), 7.84–7.73 (m, 8H, Ar), 7.22 (t, *J* = 7.5 Hz, 8H, Ar) 7.02–7.15 (m, 16H, Ar), 2.60 (q, *J* = 8 Hz, 8H, CH2), 1.26 (t, *J* = 8 Hz, 12H, CH3). ^13^C NMR (DMSO-*d*_6_) *δ* (ppm): 164.1 (amidine), 152.7 (Py), 149.2 (Py), 148.6 (Ar), 129.5 (Ar), 123.2 (Py), 122.0 (Ar), 118.0 (Ar), 28.2 (CH2), 14.5 (CH3); IR (KBr, cm^−1^): 3336 (C=NH imine), 2969 (CH3), 2934 (CH2), 1680 (NCN/C=N imine), 1606 (CN pyridine/triazene), 1482 (CH2), 1257 (triazene), 1168 (triazene), 524 (Pt–N). Anal. calcd. for C56H64N18Pt2·4HCl·2H2O: C, 43.06; H, 4.65; N 16.15. Found: C, 42.94; H, 4.62 N, 16.02.

## Cell culture

Human breast cancer MCF-7 and MDA-MB-231 cells were maintained in complete growth medium DMEM supplemented with 10 % FBS and 1 % antibiotics (penicillin/streptomycin). Cells were cultured in Costar flasks and grown at 37 °C and in the atmosphere 5 % CO_2_ to sub-confluence (90–95 %). Sub-confluent cells were treated with 0.05 % trypsin and 0.02 % EDTA in calcium free phosphate buffered saline, counted in hemocytometer and seeded in 6-well plates (Nunc) in 2 mL of growth medium (DMEM without phenol red with 10 % CPSR1). Cells, which reached about 80 % of confluency, were used for the assays.

## Cell viability assay

Cell growth was evaluated in MCF-7 and MDA-MB-231 following treatment with single or combination therapies using MTT (3-(4,5-dimethylthiazole-2-yl)-2,5-diphenyltetrazolium bromide) assay [14]. Absorbance of converted dye in living cells was measured at a wavelength of 570 nm. Cell viability of breast cancer cells cultured in the presence of ligands was calculated as a per cent of control cells.

## [^3^H]thymidine incorporation assay

The incorporation of [^3^H]thymidine into DNA was used as a measure of cell proliferation. MCF-7 and MDA-MB-231 cells were seeded in 6-well tissue culture plates at a density of 5 × 10^5^ well^−1^ in complete growth media and grown as describe above. Cells were treated with different concentration of monoclonal antibody anti-MUC1 GP1.4, cisplatin and Pt12 alone and in mixture with anti-MUC1. Cells were incubated with compounds for 24 h at 37 °C before 0.5 μC, [^3^H]thymidine was added to each well for 4 h period to measure the incorporation of radioactive component into the DNA. Radioactivity was quantitated in a scintillation counter. [^3^H]thymidine incorporation was expressed as dpm well^−1^. Each experiment was repeated at least three times.

## Collagen production

Incorporation of radioactive precursor into proteins was measured after labeling of the cells in growth medium with varying concentrations of monoclonal antibody anti-MUC1 GP1.4, Pt12, cisplatin alone and in mixture with anti-MUC1 for 24 h with 5-[^3^H]proline (5 μCi ml^−1^, 28 Ci mmol^−1^). Incorporation of tracer into collagen was determined by digesting proteins with purified *Clostridium histolyticum* collagenase, according to the method of Peterkofsky et al. [15]. Results are shown as combined values for cell plus medium fractions.

## Flow cytometry assessment of annexin V binding

Apoptosis was determined assessing phosphatidylserine exposure by Annexin V-FITC binding by means of the FITC Annexin V Apoptosis Detection Kit II according to the manufacturer’s instruction. Cells (10 000 cell measured) were analyzed in a flow cytometer (BD FACSCanto II flow cytometer, CA, USA). Annexin V bound with high affinity to phosphatidylserine and thus could be used to identify cells in all stages of the programmed cell death [16]. Propidium iodide (PI) exclusively stained cells with a disrupted cell membrane and could be used to identify late apoptotic and dead cells. Cells cultured in a drug-free medium were used as controls. Optimal parameter settings were found using a positive control (cells incubated with 3 % formaldehyde in buffer during 30 min on ice). Forward scatter (FS) and side scatter (SC) signals were detected on a logarithmic scale histogram. FITC was detected in the FL1 channel (FL1 539; Threshold–value 52). Analysis was performed using the BD FACSCanto II flow cytometer, and results were analyzed with FACSDiva software (both from BD Bioscences Systems, San Jose, CA, USA).

## Analysis of mitochondrial membrane potential

Disruption of the mitochondrial membrane potential (MMP) was assessed using the lipophilic cationic probe 5,5′,6,6′-tetrachloro-1,1′,3,3′-tetraethylbenzimidazolcarbocyanine iodide (JC-1 MitoScreen kit; BD Biosciences) as described previously [17]. Briefly, unfixed cells were washed and resuspended in PBS supplemented with JC-1. Cells were then incubated for 15 min at room temperature in the dark, washed, and resuspended in PBS for immediate BD FACSCanto II flow cytometry analysis. The percentage of cells with disrupted MMP was calculated in the FACSDiva software (both from BD Bioscences Systems, San Jose, CA, USA).

## DNA fragmentation assay

DNA fragmentation was examined by the terminal deoxynucleotidyltransferase (TdT)-mediated dUTP nick end labeling (TUNEL) method using a commercial assay kit (APO-Direct Kit; BD Pharmingen, San Diego, CA, USA). After treatment, cells were fixed with 1 % paraformaldehyde in PBS (4 °C, 30 min), washed in PBS, and permeabilized with ice cold 70 % ethanol. The Apo-Direct Kit-TUNEL assay was performed as described by the manufacturer. Cells were analyzed in a BD FACSCanto II flow cytometer. In total, 10,000 events were collected per test sample. The results were analyzed in FACSDiva software (both from BD Biosciences Systems, San Jose, CA, USA). The percentages of TUNEL negative and positive cells were presented.

## Statistical analysis

Experimental data were represented as mean ± SD since each experiment was repeated three times. Statistical analysis was performed using Statistica 6.0 (StatSoft, USA).

## Results

**Fig. 2:**
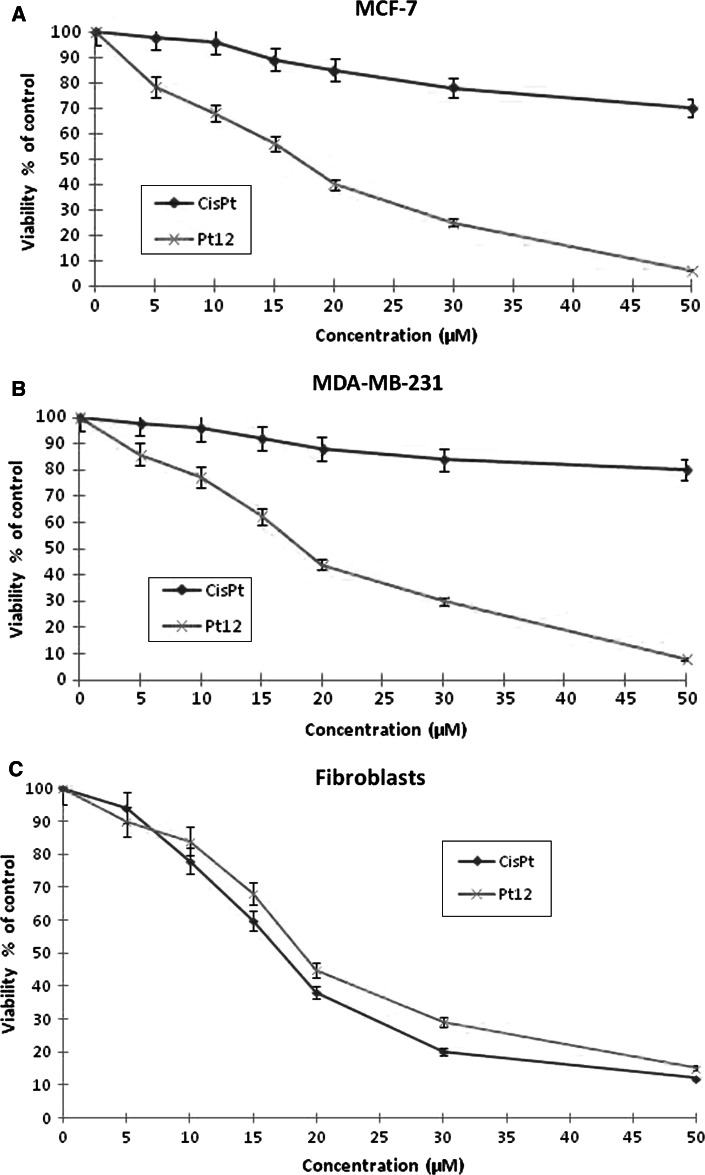
Viability of MCF-7 (**a**), MDA-MB-231 (**b**) and fibroblasts (**c**) treated for 24 h with different concentrations of Pt12 and cisplatin (cisPt). Mean values ± SD from three independent experiments (*n* = 3) done in duplicate are presented

**Fig. 3:**
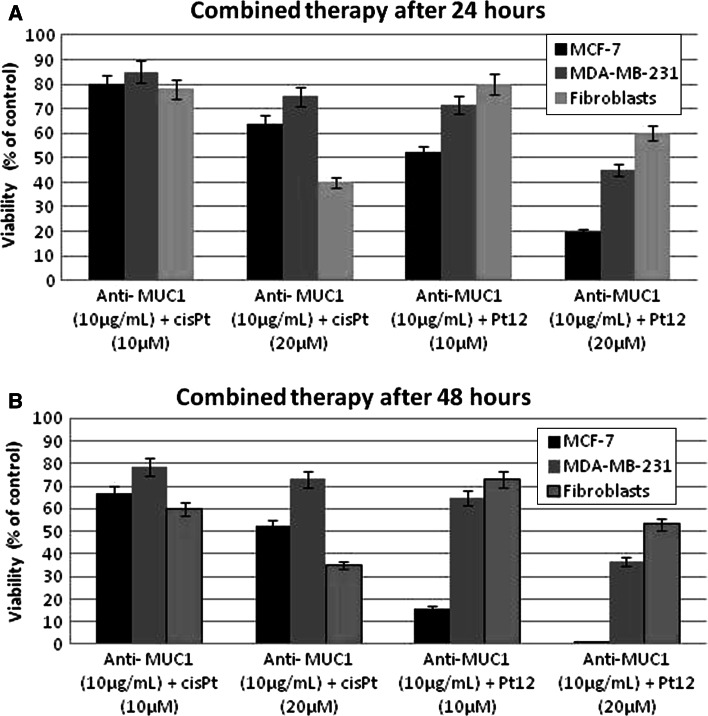
Viability of MCF-7, MDA-MB-231, and fibroblasts treated for 24 h (**a**) and 48 h (**b**) with different concentrations of combination of Pt12 + anti-MUC and cisplatin + anti-MUC1. Mean values ± SD from three independent experiments (*n* = 3) done in duplicate are presented

The cell viability of breast cancer cells and fibroblasts was measured by the method of Carmichael et al. [14], in order to compare cytotoxicity of Pt12, cisplatin, Pt12 with anti-MUC1 and cisplatin with anti-MUC1 (Figs. 2, 3). Pt12 and cisplatin decreased the number of viable cells in both breast cancer cell lines MCF-7 (Fig. 2a) and MDA-MB-231 (Fig. 2b) in dose-dependent manner, but Pt12 was more cytotoxic agent, with IC_50_ values of 17 ± 2 and 18 ± 2 μM, respectively, and compared to 82 ± 2 and 93 ± 2 μM for cisplatin. Cisplatin was more cytotoxic than Pt12 in fibroblasts, which were used as a control cell line (Fig. 2c).

Pt12 with anti-MUC1 was more effective in decreasing the viability of human breast cancer cells compared to the therapy cisplatin with anti-MUC1 (Fig. 3). The combined treatment of Pt12 (10 μM) with anti-MUC1 (10 μg mL^−1^) decreased the number of viable cells to 52 % in MCF-7 cells and 71.8 % in MDA-MB-231 cells (Fig. 3a). It was more cytotoxic compared to cisplatin (10 μM) with anti-MUC1 (10 μg mL^−1^), which reduced the number of viable MCF-7 cells to 79.8 % and MDA-MB-231 cells to 85.1 % (Fig. 3a). The anti-MUC1 at a concentration of 10 μg mL^−1^ decreased the number of viable cells to 83.0 % in MCF-7 cells and 93.0 % in MDA-MB-231 cells, respectively (data not shown). After 48 h of incubation with agents the viability of breast cancer cells significantly decreased (Fig. 3b).

**Fig. 4:**
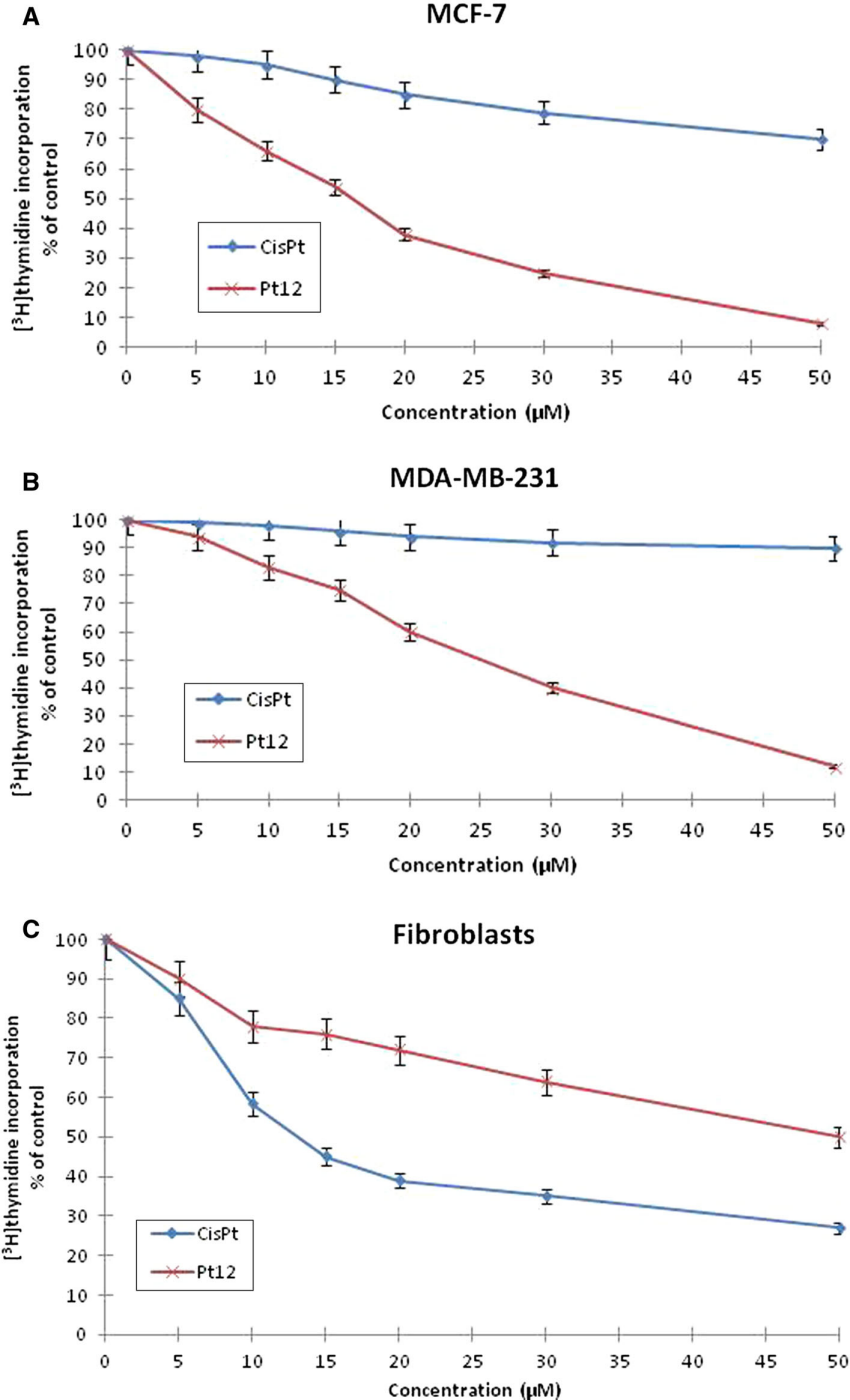
Antiproliferative effects of Pt12 and cisplatin in cultured MCF-7 (**a**), MDA-MB-231 (**b**) and fibroblasts (c) cells as measured by inhibition of [^3^H]thymidine incorporation into DNA. Mean values ± SD from three independent experiments (*n* = 3) done in duplicate are presented

**Fig. 5:**
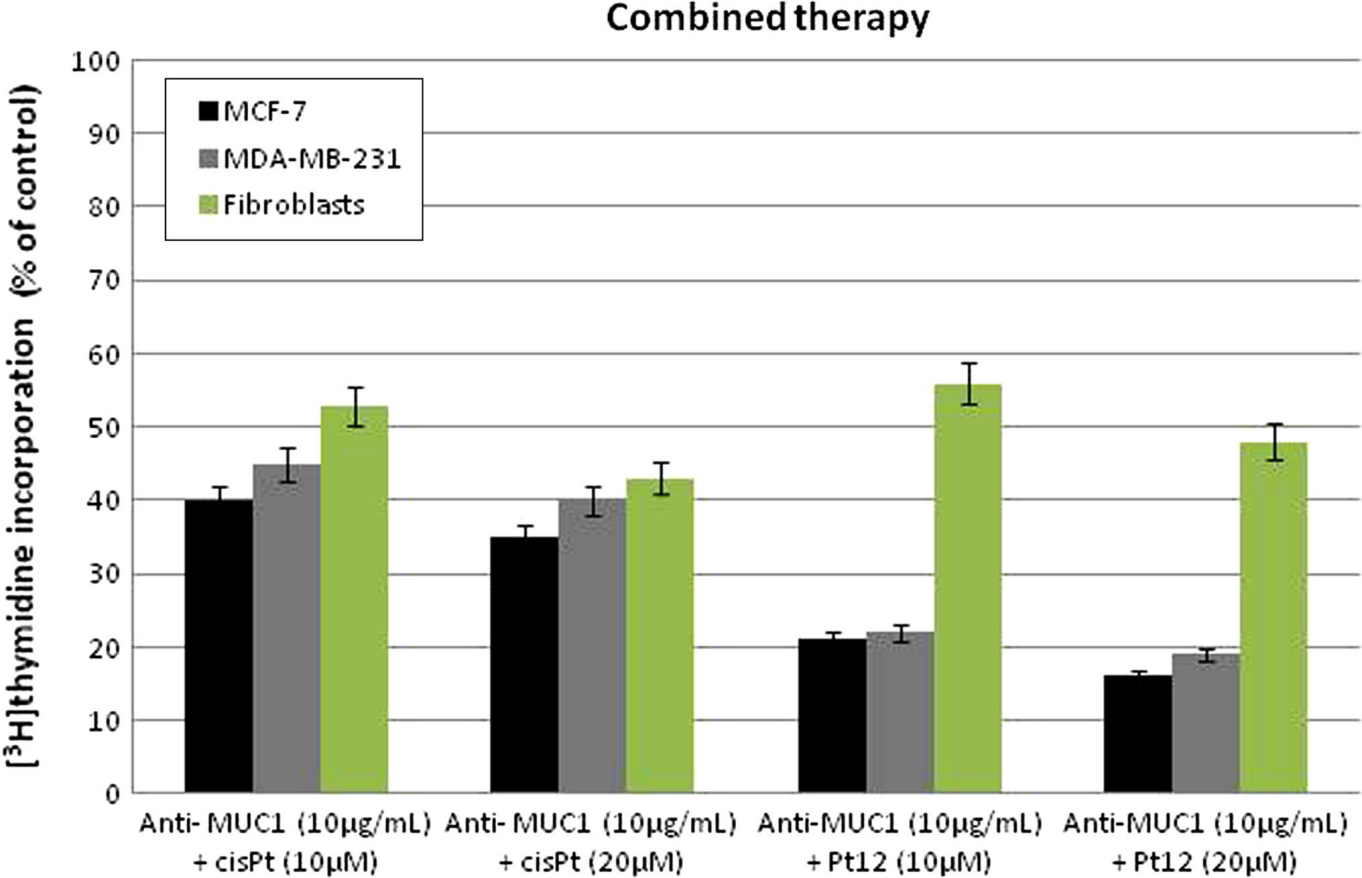
Antiproliferative effects of combined treatment of Pt12 + anti-MUC1 and cisplatin + anti-MUC1 in cultured breast cancer MCF-7, MDA-MB-231 cells, and fibroblasts as measured by inhibition of [^3^H]thymidine incorporation into DNA. Mean values ± SD from three independent experiments (*n* = 3) done in duplicate are presented

DNA synthesis was measured in the presence of Pt12, cisplatin, Pt12 with anti-MUC1 and cisplatin with anti-MUC1 to find out whether the cell viability was inhibited due to decreased cell proliferation (Figs. 4, 5). All of the study compounds showed the concentration-dependent activity, yet with different potency. Furthermore, the profiles of DNA synthesis obtained were similar between MCF-7 and MDA-MB-231. The concentrations of Pt12 required to inhibit [^3^H]thymidine incorporation into DNA by 50 % (IC_50_) in MDA-MB-231 after 24 h was found to be 25 ± 2 μM, suggesting higher cytotoxic potency Pt12 compared to cisplatin (IC_50_ = 86 ± 2 μM). The concentrations of Pt12 and cisplatin required for a 50 % reduction in [^3^H]thymidine incorporation into DNA in breast cancer MCF-7 (IC_50_) after 24 h were found to be 17 ± 2 and 98 ± 2 μM, respectively (Fig. 4a, b). Cisplatin strongly reduced [^3^H]thymidine incorporation into DNA compared to Pt12 in control cell line (Fig. 4c).

The combined treatment Pt12 (10 μM) with anti-MUC1 (10 μg mL^−1^) reduced [^3^H]thymidine incorporation into DNA in breast cancer MCF-7 after 24 h to 21.0 % in MCF-7 cells and 22.0 % in MDA-MB-231 cells (Fig. 5). Cisplatin (10 μM) with anti-MUC1 (10 μg mL^−1^) inhibited [^3^H]thymidine incorporation into DNA to 40.0 % in MCF-7 cells and 45.0 % in MDA-MB-231 cells (Fig. 5). The anti-MUC1 at a concentration of 10 μg mL^−1^ inhibited [^3^H]thymidine incorporation into DNA to 71.4 % in MCF-7 cells and 90.9 % in MDA-MB-231 cells, respectively (data not shown).

**Fig. 6:**
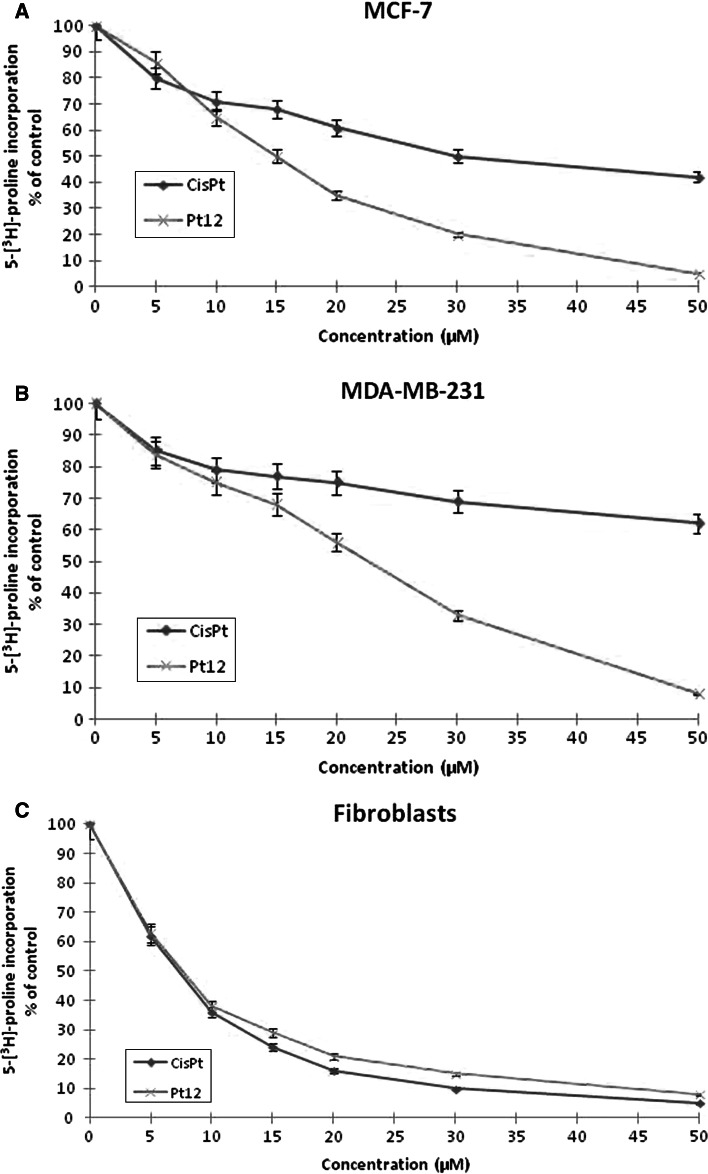
Collagen synthesis as measured by inhibition of 5-[^3^H]-proline incorporation into proteins in breast cancer MCF-7 (**a**), MDA-MB-231 (**b**) and fibroblasts (**c**) cultured for 24 h in the presence of different concentration of Pt12 and cisplatin. Mean values ± SD from three independent experiments (*n* = 3) done in duplicate are presented

One of the characteristic features of breast cancer cells is a deregulation of their interaction with extracellular matrix proteins. Therefore, changes in the quantity, structure, and distribution of collagens caused by anticancer agents may affect the metabolism and function of human breast cancer cells. Collagen biosynthesis was measured in MCF-7, MDA-MB231 breast cancer cells, and fibroblasts treated with various concentrations of cisplatin and Pt12 for 24 h. As shown in Fig. 6a, b in both cell lines, Pt12 was found to be a more effective inhibitor of collagen biosynthesis than cisplatin. The inhibitory effect was dose-dependent. IC_50_ for cisplatin and Pt12 (in MCF-7: 30 ± 2 and 15 ± 1 μM, in MDA-MB231: 64 ± 2 and 22 ± 2 μM, respectively) showed a specific inhibitory effect of Pt12 on collagen biosynthesis. As shown in 6C, cisplatin was more cytotoxic agent than newly synthesized Pt12 in control fibroblasts.

**Fig. 7:**
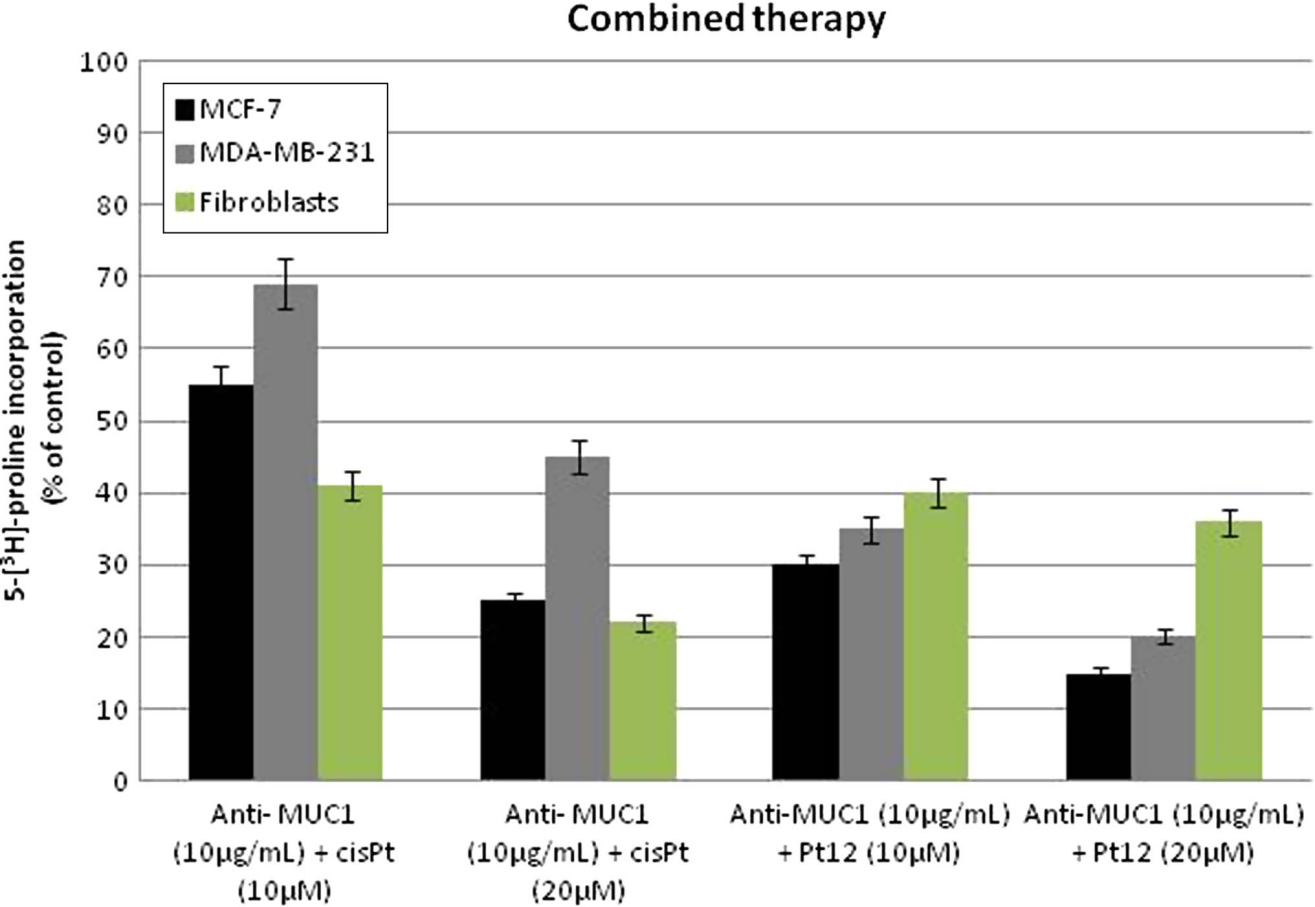
Collagen synthesis as measured by inhibition of 5-[^3^H]-proline incorporation into proteins in breast cancer MCF-7, MDA-MB-231 and fibroblasts cultured for 24 h in the presence of Pt12 + anti-MUC1 and cisplatin + anti-MUC1. Mean values ± SD from three independent experiments (*n* = 3) done in duplicate are presented

Pt12 with anti-MUC1 was found to be a more effective inhibitor of collagen biosynthesis compared to cisplatin with anti-MUC1 in the same concentration. Pt12 with anti-MUC1 inhibited 5-[^3^H]-proline incorporation into proteins to 30.0 % in MCF-7 cells and 35.0 % in MDA-MB-231 cells whereas cisplatin and anti-MUC1 inhibited the incorporation of the radioactive proline into proteins to 55.0 % in MCF-7 cells and 69.0 % in MDA-MB-231 cells (Fig. 7a, b). The addition of anti-MUC1 to complexes of platinum increases the degree of inhibition of collagen biosynthesis. The anti-MUC1 at a concentration of 10 μg mL^−1^ inhibited 5-[^3^H]-proline incorporation into DNA to 82.0 % in MCF-7 cells and 89.0 % in MDA-MB-231 cells, respectively (data not shown). Combined treatment was found to be less effective in inhibition of collagen synthesis in control cells (Fig. 7c).

**Fig. 8:**
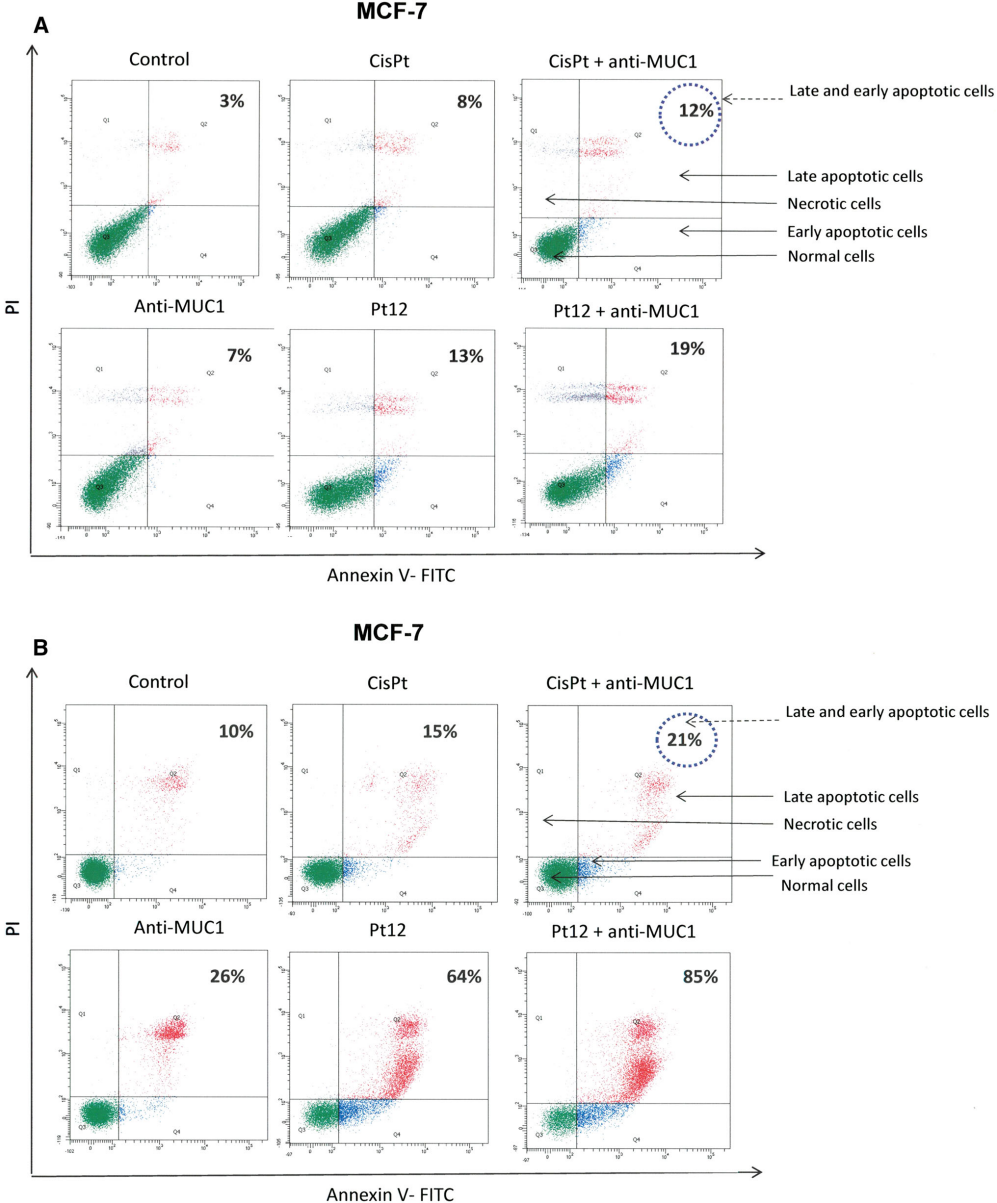
Flow cytometric analysis of breast cancer MCF-7 cells after 24 (**a**) and 48 h (**b**) of incubation with anti-MUC1 (10 μg mL^−1^), Pt12 (10 μM), Pt12 + anti-MUC1 (10 μM + 10 μg mL^−1^), cisplatin (10 μM), cisplatin + anti-MUC1 (10 μM + 10 μg mL^−1^) for 24 h and staining with Annexin V and propidium iodide (PI). Dots with Annexin V−/PI− (Q3), Annexin V+/PI− (Q4), and Annexin V+/PI+ (Q2) features represent intact, early apoptotic, and necrotic cells, respectively. Mean percentage values from three independent experiments (*n* = 3) done in duplicate are presented

The cell death was measured by flow cytometric analysis after annexin V-FITC and propidium iodide staining. The incubation of MCF-7 and MDA-MB-231 breast cancer cells with Pt12, cisplatin, Pt12 with anti-MUC1, and cisplatin with anti-MUC1 induced the visible phosphatidylserine exposure after 24 and 48 h of treatment (Fig. 8). The apoptotic effect of Pt12 was found to be stronger than that caused by cisplatin (Fig. 8). The ratio of early (lower right) and late apoptotic cells (upper right) was increased from 3.0 to 7.0 % after antibody treatment, from 3.0 to 13.0 % after Pt12 treatment, from 3.0 to 8.0 % after cisplatin treatment. The ratio of early and late apoptotic cells was increased after 24 h of combined treatment with Pt12 with anti-MUC1 (10 μM + 10 μg mL^−1^) in MCF-7 from 3.0 to 19.0 %. After combined treatment with cisplatin with anti-MUC1 used in the same doses as Pt12 with anti-MUC1 (10 μM + 10 μg mL^−1^) in MCF-7 for 24 h the ratio of early and late apoptotic cells was increased from 3.0 to 12.0 % (Fig. 8). The ratio of necrotic cells (upper left) was, however, also increased after treatment with the Pt12 with anti-MUC1 for 24 h (from 2.3 to 12.2 %) compared to treatment with cisplatin with anti-MUC1 (from 2.3 to 6.7 %). After 48 h of incubation with drugs the ratio of early and late apoptotic cells increased in all cases. We have found that the apoptotic effect of Pt12 with anti-MUC1 was stronger than evoked by cisplatin, anti-MUC1, Pt12, and cisplatin used with anti-MUC1. The ratio of early and late apoptotic cells was increased from 10.0 to 85.0 % (Fig. 8b). These results suggested that cytotoxicity of compounds against MCF-7 breast cancer cells is due to inducing apoptotic cell death as well as necrotic cell death.

**Fig. 9:**
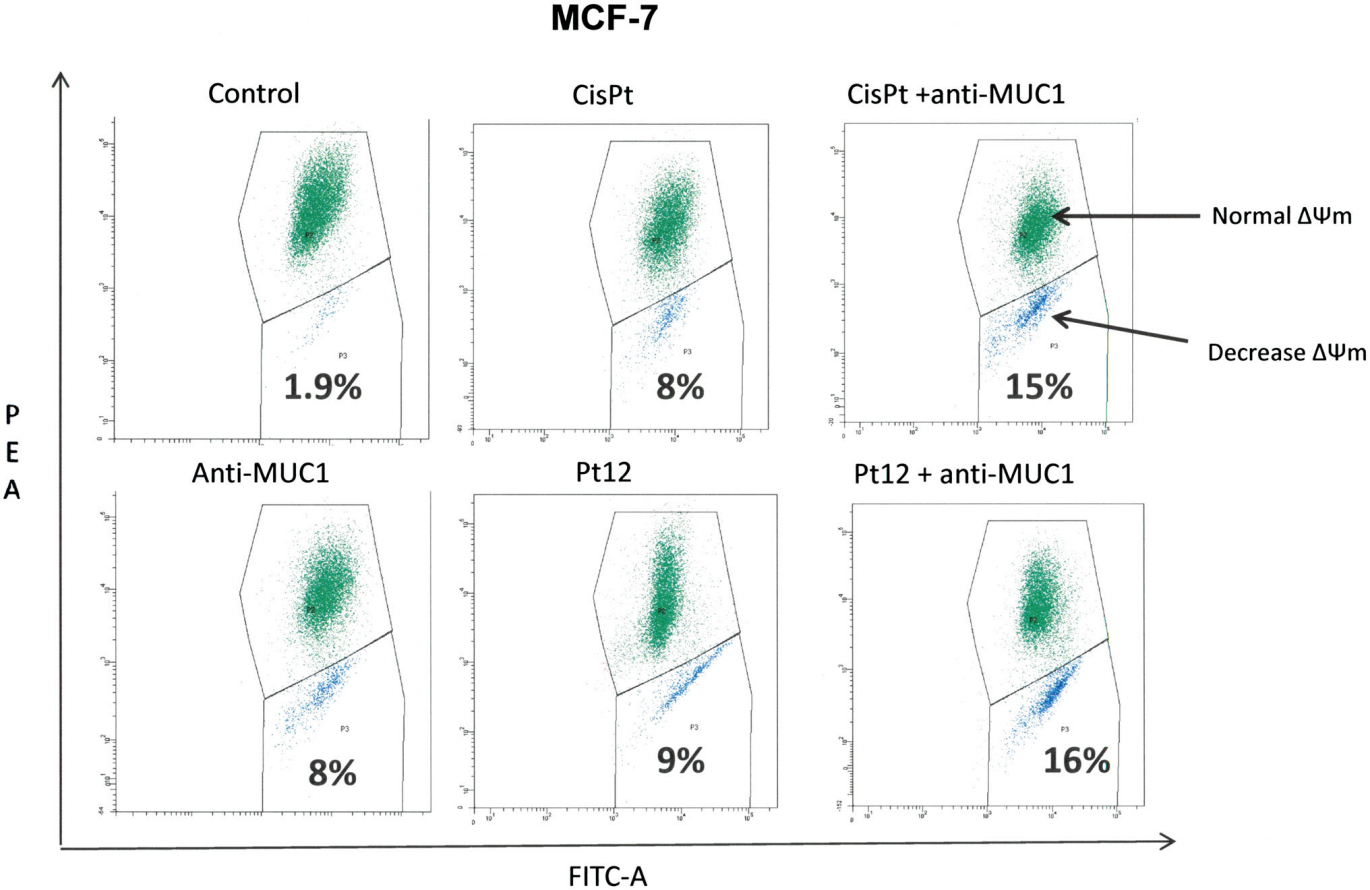
The loss of mitochondrial membrane potential of breast cancer MCF-7 cells after 24 h incubation with anti-MUC1 (10 μg mL^−1^), Pt12 (10 μM), Pt12 + anti-MUC1 (10 μM + 10 μg mL^−1^), cisplatin (10 μM), cisplatin + anti-MUC1 (10 μM + 10 μg mL^−1^) as measured JC-1 fluorescence. Mean percentage values from three independent experiments (*n* = 3) done in duplicate are presented

Mitochondria play an important role in the apoptotic process. Apoptosis proceeding through the mitochondrial pathway generally displays the permeabilization of mitochondrial membrane, in company with a dissipation of mitochondrial membrane potential (Δ*Ψ*_m_) [18]. The change of Δ*Ψ*_m_ was, therefore, determined using lipophilic cation fluorochrome JC-1 that emits green fluorescence when it is in monomer and red fluorescence when it is in aggregate. Figure 9 shows the effects of Pt12, cisplatin alone or in mixture with anti-MUC on the ΔΨ_m_ of breast cancer cells expressed as relative red-to-green fluorescence ratio, setting 100 as a benchmark (control value). The dissipation effect of Pt12 with anti-MUC1 on Δ*Ψ*_m_ is particularly conspicuous, the red-to-green fluorescence ratio at 10 μM reaches to 16 % of the control value in MCF-7 cells, suggesting that the mitochondrial membrane has been severely damaged (Fig. 9). These results are in accord with those obtained in the Annexin V/PI assay and indicate that the apoptosis induced by Pt12 with anti-MUC1 may go through the mitochondrial pathway.

**Fig. 10:**
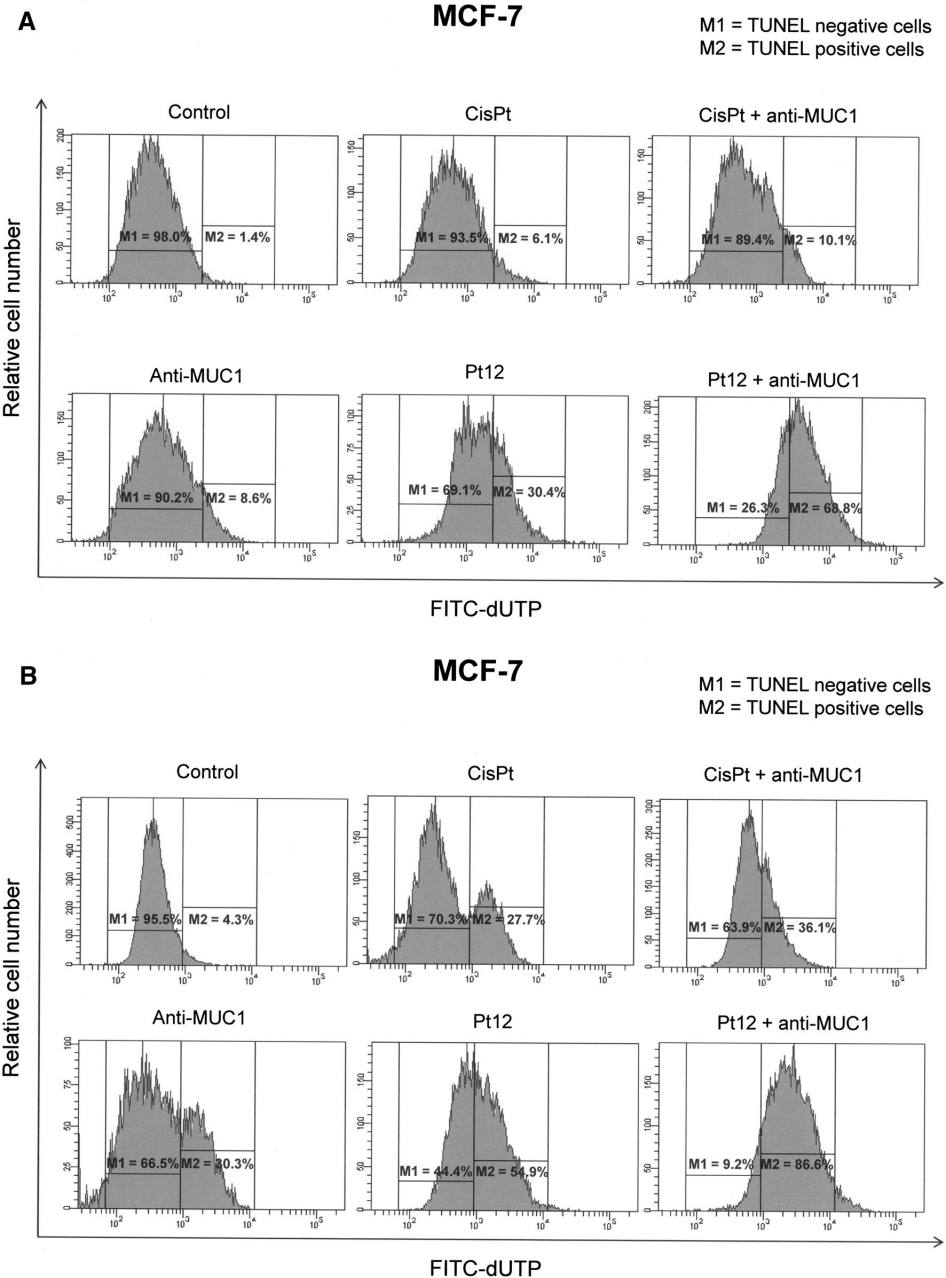
Flow cytometric analysis of DNA fragmentation of breast cancer MCF-7 cells after 24 (**a**) and 48 h (**b**) of incubation with anti-MUC1 (10 μg mL^−1^), Pt12 (10 μM), Pt12 + anti-MUC1 (10 μM + 10 μg mL^−1^), cisplatin (10 μM), cisplatin + anti-MUC1 (10 μM + 10 μg mL^−1^) using TUNEL assay. Histograms present TUNEL negative (M1-with undamaged supercoiled DNA) and TUNEL positive cells (M2-with fragmented DNA). Mean percentage values from three independent experiments (*n* = 3) done in duplicate are presented

DNA fragmentation is also associated with apoptosis. We determined whether Pt12, cisplatin, Pt12 with anti-MUC1, and cisplatin with anti-MUC1 induced DNA fragmentation in MCF-7 breast cancer cells. To detect the yield of DNA strand breaks, which are intimately associated with an apoptotic response, the TUNEL assay was performed after treating MCF-7 cells with compounds for 24 and 48 h. A highest increase in the percent of TUNEL positive cells was observed after incubation of Pt12 with anti-MUC1 in comparison to cisplatin after 24 and 48 h. Control cells cultured in normal growth media showed minimal DNA fragmentation (Fig. 10).

## Discussion

There are known research works about combined therapy with monoclonal antibody C595 and docetaxel in several ovarian cell cancers [19], but there are no data about combined therapy platinum compounds with anti-MUC1 in breast cancer cell lines. Li Wang and others checked the in vitro effect of anti-MUC1 C595 alone and in combination with docetaxel on survival of epithelial ovarian cancer (EOC) cell lines. They showed that combined treatment of docetaxel and anti-MUC1 greatly improved efficiency of cell killing and induced apoptosis in EOC cells [19]. That combination therapy was investigated in EOC mouse models. It was observed the reduction of tumor burden in dose-dependent manner. These results were consistent with previous studies in vitro [20]. Our results showed that Pt12 with anti-MUC1 is more effective than Pt12 alone or cisplatin with anti-MUC1 in breast cancer MCF-7 and MDA-MB-231 cells. The antiproliferative effect of Pt12 with anti-MUC1 was dependent on the estrogen receptor status of the breast cancer cells. Zaretsky and others discussed about the possible involvement of estrogen receptors in regulation of the MUC1 gene transcription. However, it is known that ER may regulate gene transcription also by interaction with other transcription factors (STAT, AP1, EGFR or NF-kB) without direct binding to estrogen receptors [21].

Pt12 with anti-MUC1 was proved to be a stronger inhibitor of collagen biosynthesis in breast cancer cells compared to Pt12 alone and cisplatin with anti-MUC1. One of the characteristic features of breast cancer cells is a deregulation of their interaction with extracellular matrix proteins [22]. Collagen is the most abundant component of extracellular matrix and is responsible for the maintenance of the architecture and the integrity of the connective tissue. It is known that the interaction between integrin receptors and extracellular matrix proteins, e.g. collagen, can regulate neoplastic cell attachment, migration, proliferation, progression, and survival [23]. A decreased amount of collagen in extracellular matrix is believed to enhance motility and invasion of neoplastic cells but it also contributes to the inhibition of cell growth and induction of apoptosis [24]. MUC1 can interact with EGF receptor (EGFR) and its stimulation leads to activation of multiple intracellular signaling cascades including ERK and Akt. Inhibition of ErbB (family of protein kinases) pathways with monoclonal antibodies restores normal cell proliferation and induces apoptosis in vitro [7]. Bitler and others obtained peptides which blocked the interactions between MUC1/EGFR. They proved that these peptides have antitumor properties and could be used as therapeutic agents [25].

Our experiments carried out with flow cytometry assessment of annexin V binding revealed that Pt12 with anti-MUC1 inhibited the proliferation of MCF-7 cells by increasing the number of apoptotic and necrotic cells (Fig. 8). Recent studies have shown that berenil dinuclear platinum(II) complexes induced apoptosis of breast cancer cells by extrinsic and intrinsic pathways [26]. Understanding the pathways by which Pt12 with anti-MUC1 induce cell death can provide information necessary to target specific cell death pathways in the treatment of breast cancer.

Mitochondria are considered as major players in apoptosis of mammalian cells and undergo functional and structural changes early during the death process [27]. Most apoptotic pathways converge on the mitochondria, inducing the disruption of the mitochondrial transmembrane potential Δ*Ψ*_m_. The loss of mitochondrial membrane potential Δ*Ψ*_m_ is an early and already irreversible stage of apoptosis [28]. Exposure to Pt12 with anti-MUC1 resulted decrease of mitochondrial membrane potential Δ*Ψ*_m_ in MCF-7 cells (Fig. 9). These results provide better insight into the action of Pt12 with anti-MUC1 and establish mitochondria as the major mediator of their effect in breast cancer cells. Additional studies are currently being carried out in order to determine whether Pt12 with anti-MUC1 retains its activity in other tumor cell lines.

A novel dinuclear platinum(II) complex (Pt12) used with anti-MUC1 was compared to its parent drug and cisplatin with anti-MUC1 in respect to cytotoxicity, DNA, and collagen biosynthesis in MDA-MB-231 and MCF-7 human breast cancer cells. It was found that Pt12 with anti-MUC1 was more active inhibitor of DNA and collagen synthesis as well more cytotoxic agent than Pt12 alone and cisplatin with anti-MUC1. These results indicate the use of Pt12 with anti-MUC1 may constitute a new strategy in the chemotherapy of breast cancer tumors.
